# The Aurora A-HP1γ pathway regulates gene expression and mitosis in cells from the sperm lineage

**DOI:** 10.1186/s12861-015-0073-x

**Published:** 2015-05-29

**Authors:** Phoebe H. Leonard, Adrienne Grzenda, Angela Mathison, Dean E. Morbeck, Jolene R. Fredrickson, Thiago M. de Assuncao, Trace Christensen, Jeffrey Salisbury, Ezequiel Calvo, Juan Iovanna, Charles C. Coddington, Raul Urrutia, Gwen Lomberk

**Affiliations:** Division of Reproductive Endocrinology and Infertility, Mayo Clinic, Rochester, MN 55905 USA; Department of Medicine, Mayo Clinic, Laboratory of Epigenetics and Chromatin Dynamics, Gastroenterology Research Unit, Guggenheim 10, 200 First Street SW, Rochester, MN 55905 USA; Department of Biochemistry and Molecular Biology, Mayo Clinic, Rochester, MN 55905 USA; Molecular Endocrinology and Oncology Research Center, Centre Hospitalier de l’Universite Laval (CHUL) Research Center, Quebec, QC G1V 4G2 Canada; Centre de Recherché en Cancérologie de Marseille (CRCM), Institut National de la Santé et de la Recherche Médicale (INSERM), Unité 624, Stress Cellulaire, 163 Avenue de Luminy, Case 915, Parc Scientifique et Technologique de Luminy, Marseille Cedex 9, 13288 France; Translational Epigenomics Program, Center for Individualized Medicine, Rochester, MN 55905 USA; Department of Obstetrics and Gynecology, Mayo Clinic, Rochester, MN 55905 USA

**Keywords:** Epigenetics, Heterochromatin Protein 1, HP1γ, CBX3, Gametes, Preimplantation embryo, Meiosis, Mitosis, Embryonic genome activation

## Abstract

**Background:**

HP1γ, a well-known regulator of gene expression, has been recently identified to be a target of Aurora A, a mitotic kinase which is important for both gametogenesis and embryogenesis. The purpose of this study was to define whether the Aurora A-HP1γ pathway supports cell division of gametes and/or early embryos, using western blot, immunofluorescence, immunohistochemistry, electron microscopy, shRNA-based knockdown, site-directed mutagenesis, and Affymetrix-based genome-wide expression profiles.

**Results:**

We find that the form of HP1γ phosphorylated by Aurora A, P-Ser83 HP1γ, is a passenger protein, which localizes to the spermatozoa centriole and axoneme. In addition, disruption in this pathway causes centrosomal abnormalities and aberrations in cell division. Expression profiling of male germ cell lines demonstrates that HP1γ phosphorylation is critical for the regulation of mitosis-associated gene expression networks. In female gametes, we observe that P-Ser83-HP1γ is not present in meiotic centrosomes of M2 oocytes, but after syngamy, it becomes detectable during cleavage divisions, coinciding with early embryonic genome activation.

**Conclusions:**

These results support the idea that phosphorylation of HP1γ by Aurora A plays a role in the regulation of gene expression and mitotic cell division in cells from the sperm lineage and in early embryos. Combined, this data is relevant to better understanding the function of HP1γ in reproductive biology.

**Electronic supplementary material:**

The online version of this article (doi:10.1186/s12861-015-0073-x) contains supplementary material, which is available to authorized users.

## Background

Strong experimental evidence generated during the last decade has established that epigenetic mechanisms are both necessary and required for the development and maintenance of reproductive function [1]. At the mechanistic level, epigenetic regulation primarily consists of establishing, maintaining, and reversing posttranslational modifications (chemical marks) present on DNA and histones, as well as the function of several types of both small and large non-coding RNA molecules [2]. Within a particular cell type, these epigenetic regulators function as signals that define gene expression patterns, which establish and maintain normal and diseased phenotypes. Epigenetic marks are generated by a highly specialized group of proteins responsible for their deposition (writers), interpretation (readers), and when necessary, erasure from chromatin (erasers). The HP1 family of reader proteins was one of the first discovered epigenetic regulators and most versatile of the proteins that regulate chromatin-based inheritance [3]. There are three HP1 subtypes α, β, and γ, encoded in humans by *CBX5*, *CBX1*, and *CBX3*, respectively [4]. These proteins have three domains with the N-terminal chromodomain binding to lysine 9 methylated histone marks, joined by a flexible linker region to a carboxy-terminal chromoshadow domain, which supports dimerization and interaction with other chromatin proteins [5]. HP1α and HP1β are mostly associated with transcriptionally inactive heterochromatin, whereas HP1γ is observed in both heterochromatin and transcriptionally active euchromatin [6]. Functionally, HP1 proteins regulate development through their ability to determine various cellular phenotypes by regulating entire gene expression networks at the appropriate level, time, and place. HP1 proteins are also emerging as candidate regulators of reproductive function; however, their roles in these phenomena have just begun to be elucidated. For instance, recent studies demonstrate that genetic inactivation of HP1γ in the germ line results in adult males carrying severe spermatogenic defects and growth retardation [7, 8]. Interestingly, previous work in somatic cells has shown that HP1γ is phosphorylated, acetylated, ubiquitinated, sumoylated, and methylated [9], though whether these posttranslational modifications have any impact on the biology of cells from reproductive organs remains to be defined. In the current study, we describe the phosphorylation, localization, and genome-wide regulatory functions of HP1γ in gonadal tissue, gametes, and the pre-implantation embryo. We demonstrate that phosphorylation of this protein at S83, which occurs in response to Aurora A [10], is necessary for supporting proper mitotic cell division in cells from the sperm lineage. Therefore, we conclude that phosphorylation of HP1γ, prior to meiosis, differentiation, and maturation, is necessary to maintain a viable pool of male gametes, extending our understanding of signaling cascades that regulate the function of this important chromatin protein in reproductive biology. Since drugs that target both Aurora A and the HP1γ pathway are emerging as tools for the treatment of diverse diseases including addiction and cancer, the new knowledge derived from the current study should be taken into consideration to predict potential side effects of these therapies on reproductive functions.

## Methods

### Cell lines, tissue, and gametes

Human and mouse tissue and gamete use was approved through Mayo Clinic Internal Review Board (IRB) and Institutional Animal Care and Use Committee (IACUC), respectively.

GC-1spg and GC-2spd(ts) cell lines were obtained from American Type Culture Collection and maintained according to their recommendations. GC-1spg cells were originally generated by immortalizing mouse Type B spermatogonia with SV40 that demonstrate characteristics of a stage between type B spermatogonia and primary spermatocytes [11]. GC-2spd(ts) cells were first established by transfecting mouse spermatocytes with SV40 large T antigen [12, 13]. *HP1*γ (*Cbx3*) knockdown was achieved by lentiviral shRNA (Santa Cruz Biotechnology, Inc), according to manufacturer’s instructions with puromycin selection (2 μg/mL). Quantification of mitoses was performed through immunofluorescence with γ-tubulin. For each condition, 200 mitotic cells were analyzed. Chi-square test was used for analysis with significance of p < 0.05. To determine the percentage of cells undergoing cell division, the colorimetric Mitotic Assay Kit (Active Motif) was used according to manufacturer’s instructions with normalization for cell number by Crystal Violet staining.

Human spermatozoa were obtained from a fertile sperm donor collected through masturbation after 48 hours of abstinence. The semen specimen was processed on a single 90 % layer of Isolate™ (Irvine Scientific), centrifuged, washed and re-suspended in protein-free HTF. Murine spermatozoa were obtained from 8 week old C57BL/6 mice. Epididymi were dissected and sperm were collected via the swim up method.

### Oocyte and embryo collection and culture

Four week old FVB female mice (Charles River Laboratories) were superovulated with 5 IU of intraperitoneal pregnant mare serum (NHPP) followed 48 hours later with 5 IU of intraperitoneal human chorionic gonadotropin (hCG; APP Pharmaceuticals). For oocyte collection, females were sacrificed approximately 16 hours after hCG injection and oocytes were obtained from oviducts [14]. For embryos, females were caged individually with male CF1 mice and mating confirmed by vaginal plug. Females were sacrificed and 1-cell mouse embryos were obtained from oviducts 18 hours after hCG injection. Subsequently, embryos were cultured in 25 uL microdrops of Global media (LifeGlobal) covered with 1 mL of mineral oil (Fisher Scientific). Embryos were cultured in groups of 10 at 37 °C in 6.5 % CO2 to obtain a target pH between 7.20 and 7.30 [15] and fixed at various time intervals. Day of sacrifice was considered Day 0, 18 hours after hCG injection, Day 0.5 (29.5hrs), Day 1 (42hrs), Day 3 (90hrs), Day 5 (138hrs).

### Western blot analysis, immunofluorescence, immunohistochemistry (IHC), and confocal microscopy

Western blot, immunofluorescence and confocal microscopy were all performed as previously described [9, 10].

Formalin-fixed mouse testis tissues were paraffin-embedded and sectioned (5 μm). Subsequently, IHC was performed as described [16]. Dilution of the P-Ser^83^-HP1γ antibody [9, 10] was 1:50. Light microscopy slides were incubated with biotinylated goat anti-rabbit secondary antibody (Vector Labs), followed by HRP-streptavidin (Invitrogen), and immunoreactivity was monitored with Nova Red (Vector Labs). Negative control stains were done with incubation of secondary antibody only. Sections were counterstained with hematoxylin solution.

Fluorescence images were obtained using confocal microscopy at 40X magnification using a Zeiss LSM-780 confocal microscope and images were analyzed using ZEN software. Light microscopy images were obtained at 40X magnification using a Zeiss AxioPlan2 with AxioVision software.

### *Immunoelectron microscopy*

Spermatozoa were processed for immunolabeling using a BioWave laboratory microwave (Ted Pella, Inc). Isolated spermatozoa were suspended in 1 % agar and fixed in 4 % paraformaldehyde + 0.1 % glutaraldehyde, dehydrated in a series of ethanol, and embedded in LR White resin. Sections (0.1 M) were mounted onto Ni mesh grids for immunolabeling. Grids were hydrated on drops of phosphate buffered saline (PBS) + 0.1 % TWEEN 20 followed by incubation with antigen retrieval solution (modified citrate, pH 6.1, Dako North America Inc.). Primary antibodies were incubated at room temperature in PBS + 0.1 % TWEEN 20 followed by secondary gold (10 nm) conjugate (BBI Research). Electron micrographs were acquired using a JEOL 1400 TEM operating at 80 kV (Jeol USA, Inc.).

### mRNA isolation and RT-PCR

mRNA isolation was performed on gametes and embryos using Dynabeads mRNA DIRECT Micro Kit (Invitrogen). Oocytes and embryos at different developmental stages based on experimental protocol were placed in a lysis buffer with a concentration of 5 embryos/oocytes in 20 μL and frozen at −80 °C. cDNA synthesis was performed using the Super Script VILO cDNA synthesis Kit (Invitrogen) as per manufacturer’s protocol. Real time PCR was performed using RT^2^ SYBR® Green qPCR Mastermix (Qiagen) and RT^2^ qPCR Primers (Qiagen) on the Bio-Rad CFX96 system. Fold changes and standard error of the means (S.E.M.) were calculated using Bio-Rad CFX manager or SABioscience’s RT2 Profiler PCR Array Data Analysis software. Relative levels were normalized using ΔΔCt to GAPDH. Fold-change was calculated relative to Day 0 (Day 0 = 1).

### Recombinant adenovirus and whole-genome expression profiling

Epitope-tagged (6XHis-Xpress™) wild type HP1γ, S83A or S83D as well as empty vector (EV, Ad5CMV), were generated as described [10]. GC1-HP1γ knockdown cells were infected with recombinant adenoviruses at a multiplicity of infection (MOI) of 1:200 for 48 hours prior to RNA isolation. Global gene expression profiling was carried out at the Microarrays Facility of the Research Center of Laval University CRCHUL utilizing the Affymetrix Mouse Gene 2.0 ST arrays, as previously described [10]. A threshold of log2 fold change ±1.25 and a p-value < 0.05 were used to select significantly altered genes. A threshold of log2 fold change between −2 and 2 and a p-value > 0.95 were used to identify unchanged genes. Data analysis, hierarchical clustering, and ontology were performed as previously described [10]. Selected probes and their fold changes were loaded into Ingenuity Pathways Analysis Software (IPA; Ingenuity Systems) for annotation and network enrichment/analysis. Semantic relationship analysis with IPA generated significant networks of well-characterized pathways with the satisfaction of Fisher’s Exact Test. A subset of genes was validated by qPCR (Additional file 1: Fig. S1) as previously described [10, 16].

## Results

### Function of HP1γ phosphorylation during mitotic cell division in male germ cells

**Fig. 1:**
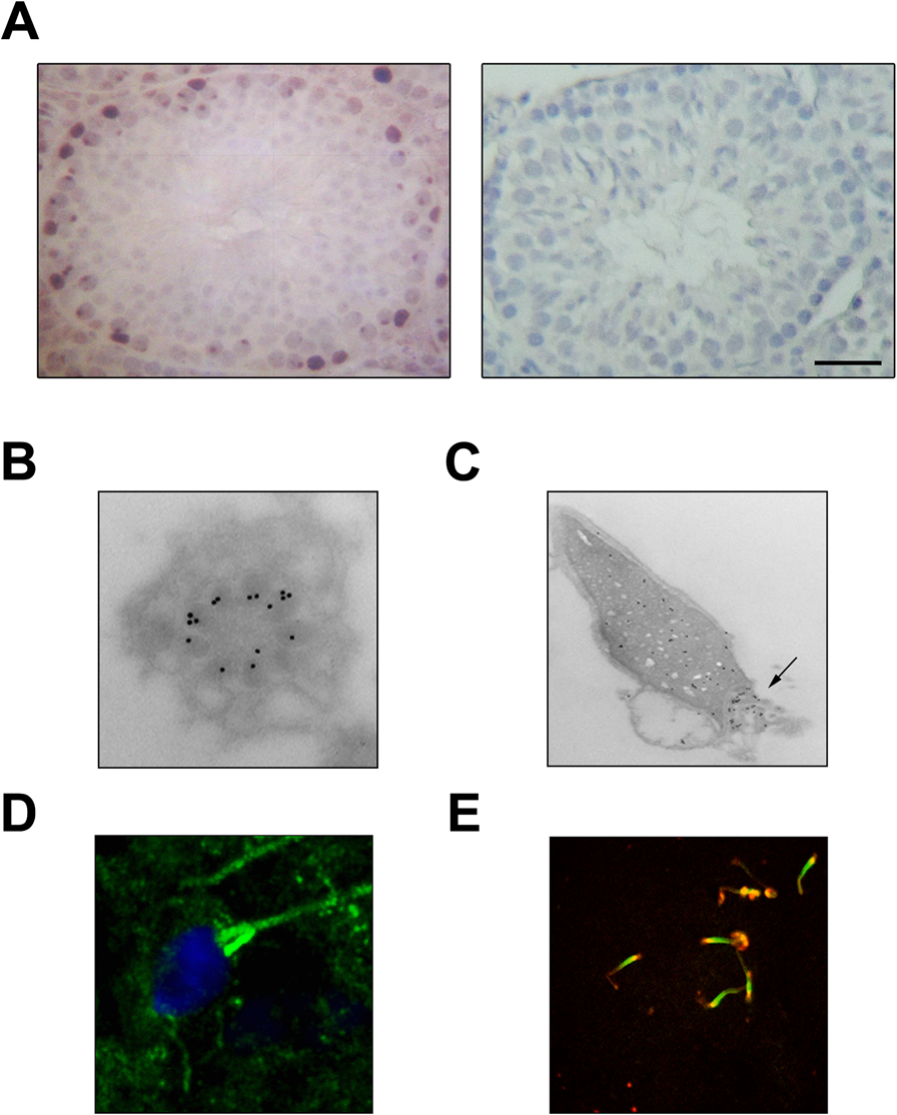
P-S83-HP1γ is present in testicular tissue and spermatozoa. **a**. P-S83-HP1γ in normal mouse testicular tissue. Immunohistochemistry of P-S83-HP1γ (left panel) reveals that the cell population near the basal lamina, typically undergoing high levels of proliferation, is the most highly P-S83-HP1γ positive. Right panel demonstrates negative control of immunohistochemistry staining with secondary antibody only. Scale bar represents 50 μM. **b** and **c**. P-S83-HP1γ localization in spermatozoa by electron microscopy. Immunogold electron microscopy depicting mature human spermatozoa. P-S83-HP1γ was concentrated in the centriole **(b)** and mid-piece (**c**, arrow) of the spermatozoa. **d** and **e**. P-S83-HP1γ localization spermatozoa by immunofluorescence. Representative image for protein localization of human spermatozoa with immunofluorescence. Localization of P-S83-HP1γ is observed adjacent to the sperm nuclei (counterstained with DAPI in **d)**, corresponding to the mid-piece of mature human sperm. This signal also co-localizes with centrin-2 (red in **e**), structural component of the centrosome located in the mid-piece

Unexpectedly, phenotypic examinations in mice carrying a gene trap that disrupts the *HP1*γ gene, initially generated for experiments that did not seek to necessarily shed light into reproductive biology, demonstrated that alterations in this chromatin protein result in azoospermia [8]. However, there is a paucity of data on how this protein is regulated to support the development and maturation of the sperm lineage. Recent studies in our laboratory have demonstrated in somatic cells that HP1γ phosphorylation at Ser^83^ is catalyzed by Aurora A [10], a kinase that plays a significant role in supporting proliferation throughout development [17]. Thus, to begin filling this important knowledge gap, we initially studied the localization specifically of the Ser^83^-phosphorylated form of HP1γ in mouse testis sections using immunohistochemistry. Notably, we found that P-Ser^83^-HP1γ localizes most strongly within cells along the basement membrane of seminiferous tubules, where mitotic spermatogonia and pre-leptotene spermatocytes reside (Fig. 1a). In order to determine whether P-Ser^83^-HP1γ could be detected in later stages of spermatogenesis, we performed higher resolution imaging on mature sperm by electron microscopy, which revealed that this phosphorylated subpopulation of HP1γ is stored primarily in the centriole and mid-piece region at the base of the sperm nucleus (Fig. 1b and c). This localization was further confirmed by immunofluorescence, which clearly showed that the signal for phosphorylated HP1γ was not localized within the sperm nuclei, as shown by DAPI counterstain, but rather coincided with the mid-piece (Fig. 1d). In addition, P-Ser^83^-HP1γ colocalized with Centrin-2, a structural component of the centrosome, which is located within the sperm mid-piece (Fig. 1e). This localization of P-Ser^83^-HP1γ is congruent with the fact that the kinase responsible for this modification, namely Aurora A, has also been shown to be localized to centrioles [18].

**Fig. 2:**
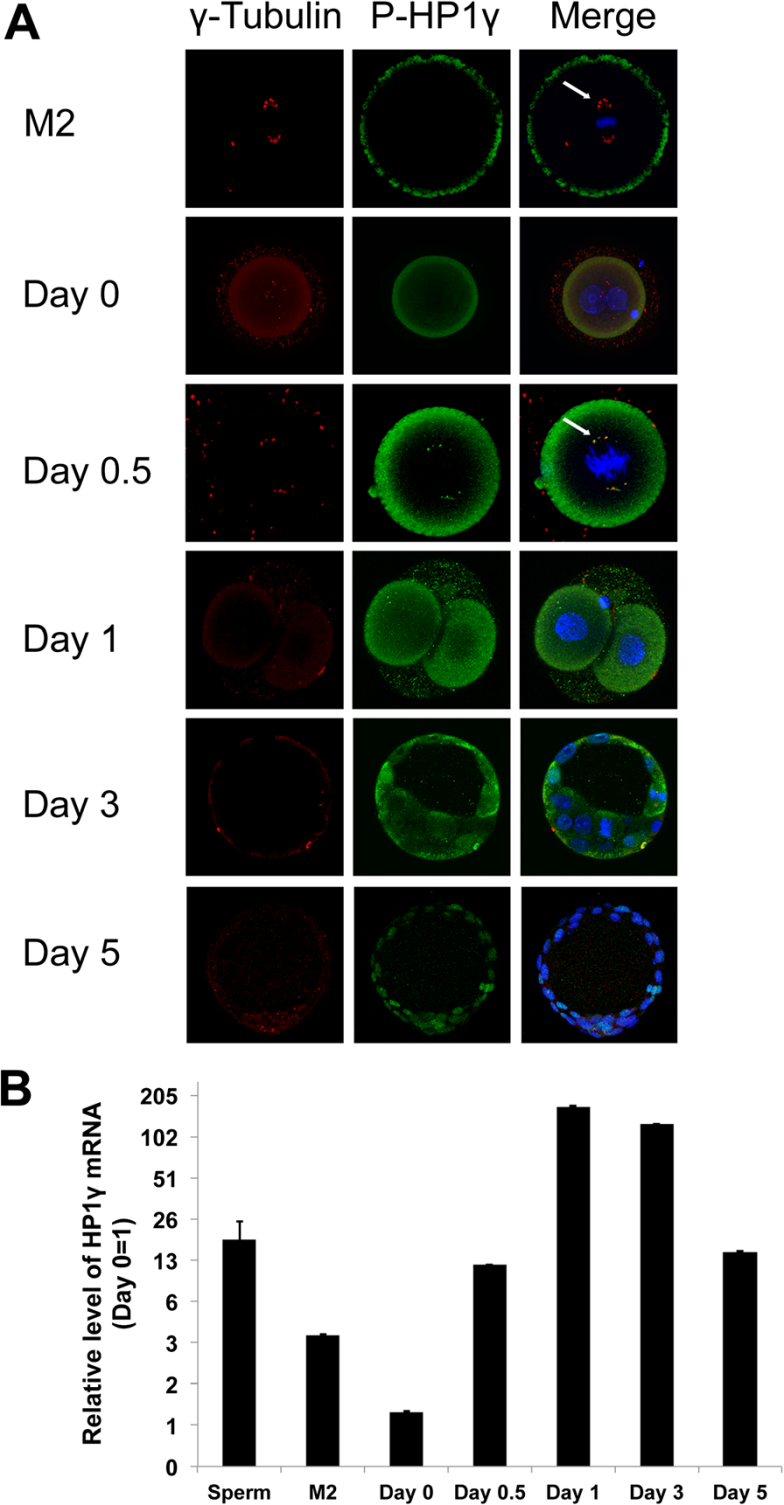
Spatiotemporal phosphorylation and expression of HP1γ increases during early embryonic development. **a**. Immunofluorescence of P-S83-HP1γ in mouse oocytes and embryos. Immunofluorescence depicting P-S83-HP1γ (green), γ Tubulin (red) and DAPI DNA staining (blue). P-S83-HP1γ colocalizes to centrosomes at the time of mouse early embryonic genome activation (Day 0.5; arrow) and maintains this location through the remainder of mitotic divisions. After syngamy this protein then localizes to its euchromatic location during interphase in cells in the preimplantation embryo.**b**. HP1γ mRNA expression during early embryonic development. mRNA from mouse oocytes and embryos were analyzed by Q-PCR. Relative expression was normalized using ΔΔCt for *HP1*γ levels to *GAPDH*. Lowest value in each analysis (Day 0) was normalized as 1 with fold changes depicted in logarithmic units on the Y-axis. Error bars represent S.E.M. Time course of embryos post HCG injection was Day 0 (18 hrs), Day 0.5 (29.5 hrs), Day 1 (42 hrs), Day 3 (90 hrs), Day 5 (138 hrs)

We subsequently asked whether the role of P-Ser^83^-HP1γ is an exclusive feature of male cell division or it is also found in oocytes and, after fertilization, during mitosis in early embryos. Interestingly, we found that M2 oocytes did not demonstrate localization of P-Ser^83^-HP1γ at spindle poles (Fig. 2a). Similarly, newly fertilized embryos, which do not yet exhibit pronuclear syngamy (Day 0), did not display P-Ser^83^-HP1γ localization to euchromatin as previously described in somatic cells [9]. However, embryos undergoing the first mitotic cell division transitioning from pronuclear syngamy (Day 0.5) to the 2-cell stage (Day 1) demonstrated localization of P-Ser^83^-HP1γ at the spindle poles, colocalizing with γ-tubulin. This event coincides with mouse early embryonic genome activation [19]. This localization was maintained in mitotic cells from the first division through the late blastocyst stage. Day 3 early blastocysts (approximately 32 cells) and day 5 late blastocysts (>100 cells) also demonstrated localization of P-Ser^83^-HP1γ to its euchromatic location (Fig. 2a). These experiments were complemented with quantitative PCR, which demonstrated that *HP1*γ transcript was 18.1-fold higher (±6.3-fold) in spermatozoa and 3.6-fold higher (±0.03-fold) in M2 oocytes than in the newly fertilized, pronuclear embryo (day 0) when normalized to *GAPDH* levels (Fig. 2b). There was a 11.8-fold increase (±0.01-fold) in *HP1*γ expression at the day 0.5 pronuclear syngamy stage compared with the day 0 pronuclear embryo before syngamy and a 168.3-fold increase (±1.4-fold) at the 2-cell stage (day 1). However, *HP1*γ expression levels decreased after this point through the early blastocyst (day 3, 125.1 ± 0.75-fold compared to day 0) and late blastocyst (day 5, 14.7 ± 0.07-fold compared to day 0) stages (Fig. 2b). Thus, combined, these results suggest that phosphorylation of HP1γ at Ser^83^ plays a key role in mitotic cell division in the male germ line and early embryonic genome activation, which is likely contributed by the sperm.

### Genetic inactivation of HP1γ in cultured male germ cell lines leads to mitotic aberrations

**Fig. 3:**
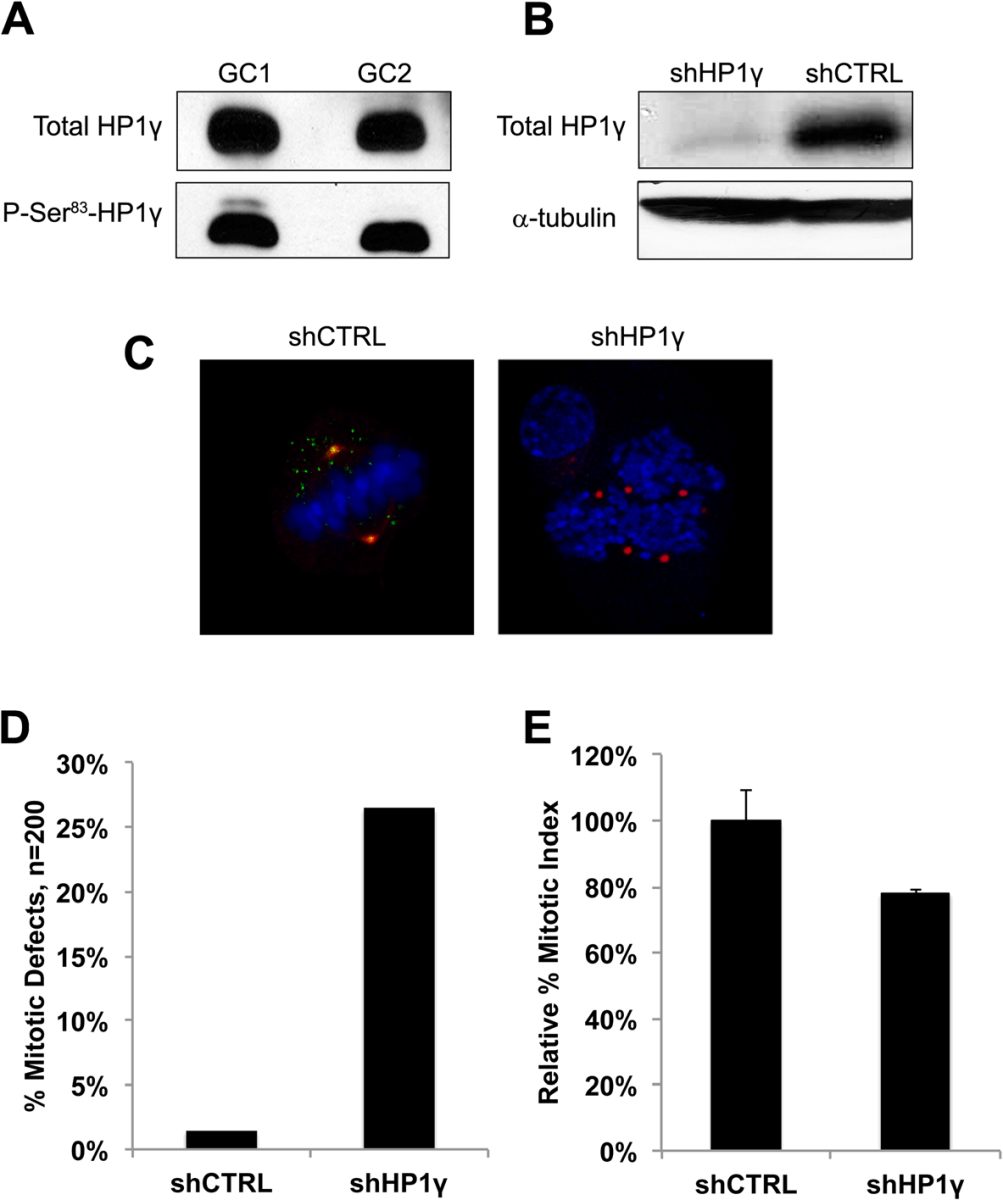
Knockdown of HP1γ in germ cells results in mitotic abnormalities. **a**. Levels of P-S83-HP1γ in GC1 and GC2 cell lines. High levels of HP1γ and its phosphorylated Ser83 form are found in the cell lines, GC1 and GC2, by Western Blot. **b**. Knockdown of *HP1*γ in GC1 cells. shRNA-mediated knockdown of *HP1*γ results in reduction of HP1γ protein levels as shown by western blot. α-tubulin is used as loading control. **c**. Immunofluorescence of *HP1*γ knockdown in GC1 cells. Immunofluorescence of control cells (shCTRL) shows colocalization of P-S83-HP1γ staining in green and γ-tubulin in red, creating a yellow signal in the overlay. DNA is counterstained with DAPI (blue). ShRNA knockdown of *HP1*γ abolishes P-S83-HP1γ staining (loss of green signal) and results in centrosomal abnormalities compared with control cells. A representative shHP1γ cell is shown where centrosomes are labeled by γ-tubulin staining (red) to demonstrate aberrant spindle pole number and localization during mitosis. **d**. Quantification of mitotic abnormalities. Quantification reveals a significantly high rate of centrosomal abnormalities in the shHP1γ cells vs shCTRL, 26.5 % and 1.5 % respectively. **e**. *HP1*γ knockdown in GC1 cells results in decreased cell division. Mitotic index assay confirms that shHP1γ cells have decreased cell division compared to shCTRL cells (78.1 % ± 1.3 %; normalized to shCTRL), likely as a result of these mitotic abnormalities

Using immortalized mouse male germ cell lines (GC1 and GC2), well-suited models for functional studies on the sperm lineage corresponding to the pre-meiotic spermatogenic cell population [11, 13], for which we observed the highest P-Ser^83^-HP1γ levels, we first measured the protein levels of both HP1γ and its phosphorylated Ser^83^ form by western blot. Fig. 3a demonstrates that indeed these proteins can be readily detected, leading us to proceed with experiments based on the genetic inactivation of HP1γ. For this purpose, we proceeded to knockdown HP1γ using stable lentiviral shRNA, which achieved an approximate 90 % reduction in protein levels as demonstrated by western blot analyses (Fig. 3b). In addition, examination of knockdown cells by immunofluorescence further confirmed decrease of HP1γ and P-Ser^83^-HP1γ staining in cells transfected with HP1γ-specific shRNA. More importantly, phenotypic examination of these cells determined that the genetic inactivation of HP1γ results in mitotic defects, which include centrosome abnormalities, multipolar spindles, and unorganized chromosomes (Fig. 3c) compared with control. Quantitative analyses revealed that these abnormalities were significantly induced by HP1γ knockdown (26.5 %) when compared to scrambled control shRNA (shCTRL) cells (Fig. 3d; 1.5 %; n = 200 in each group). Concordantly, the shHP1γ cell population also demonstrated decreased cell division compared to shCTRL cells, as measured by mitotic index assay (Fig. 3e; 78.1 % ± 1.3 % of shCTRL). Thus, these results demonstrate that normal levels of HP1γ are necessary to maintain normal mitotic cell division in pre-meiotic cells from the sperm lineage, a finding that is congruent with our immunohistochemical observations.

### HP1γ regulates gene expression networks that are key for supporting normal spermatogenesis

**Fig. 4:**
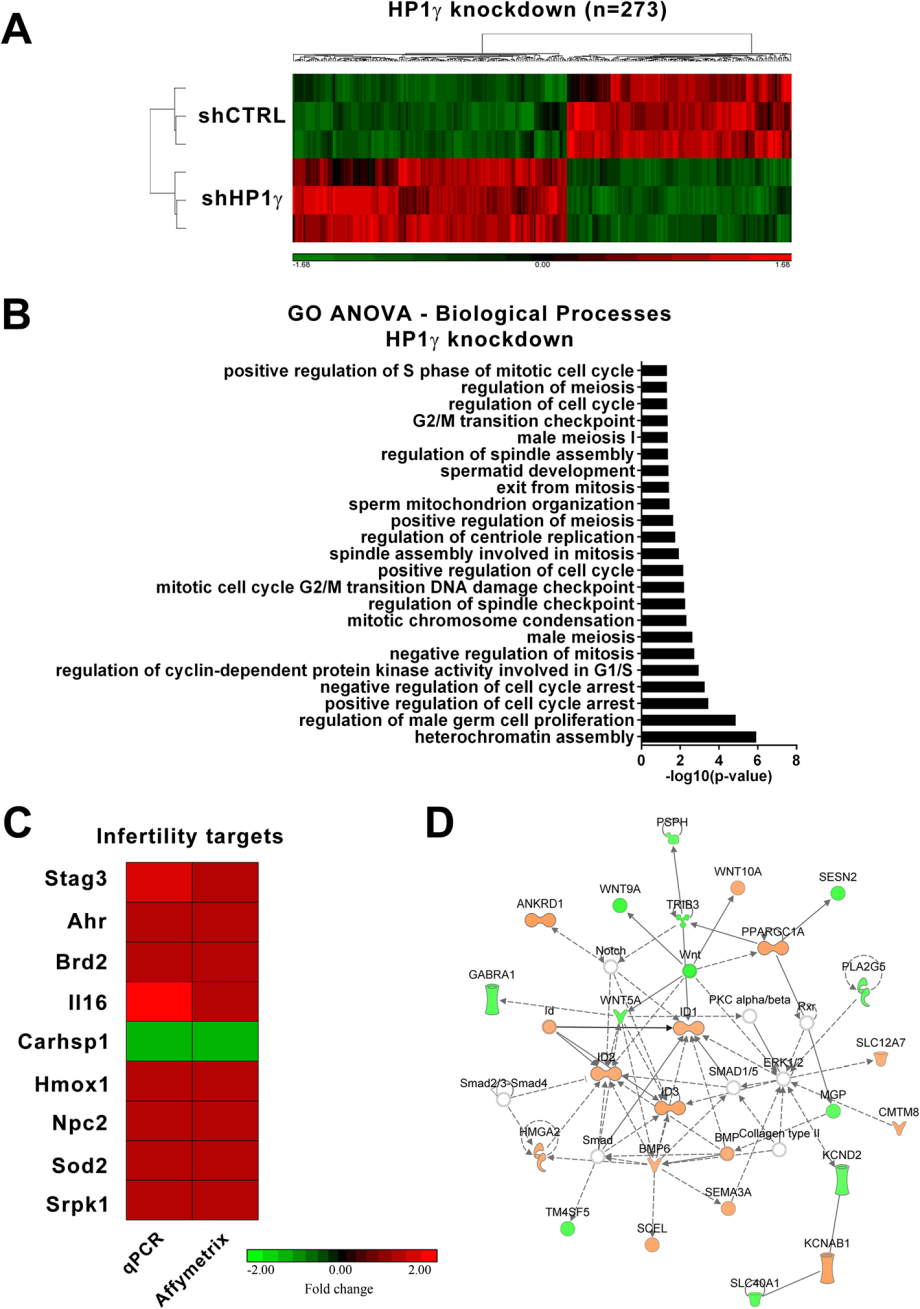
Knockdown of *HP1*γ in male germ cells impacts processes related to mitosis and meiosis. **a**. Affymetrix whole genome gene expression analysis was performed on GC1 *HP1*γ knockdown cells compared to scrambled shRNA control cells. 273 genes targets are significantly (fold change ±1.25, p < 0.005) activated or repressed in the absence of HP1γ. **b**. Gene Ontology (GO) ANOVA analysis of the 273 targets was performed and revealed significant (p < 0.05) enrichment of mitosis and meiosis associated processes, as well as processes involved in differentiation. **c**. qPCR validation of a subset of identified knockdown targets with known function in male fertility is shown. Fold change of shHP1γ compared to shCTRL expression is represented on a scale of ±2 and shown next to the corresponding Affymetrix data. **d**. The top-scoring Ingenuity-based network analysis network is significantly (p < 0.05) associated with cellular development, gene expression, and cell cycle

Since the major biochemical function of HP1γ is to regulate gene expression, we next examined the effects of this protein on genome-wide expression profiling that may influence spermatogenesis. For this purpose, we utilized our GC1 cell line stably expressing an HP1γ shRNA knockdown construct. When compared to control shRNA cells (Fig. 4a), the genetic inactivation of HP1γ resulted in 273 genes being significantly upregulated or downregulated. Further processing of this data using a Gene Ontology (GO) ANOVA analysis demonstrated that HP1γ knockdown significantly impacted biological processes involved in sperm development (p < 0.05) (Fig. 4b). This relationship was apparent by the differential regulation of gene targets involved in both mitosis and meiosis-related processes such as those ontologically-related to the regulation of the cell cycle and mitosis (Additional file 2: Table S1, Fig. 4b). To validate these results, we used real time quantitative PCR to measure the expression of a subset of spermatogenesis targets identified as significant by the Affymetrix analysis (Fig. 4c). These experiments sought to validate changes in the expression of genes with the following associated processes: meiosis (*Stag3*), spermatogenesis (*Brd2*), cell motility (*Il16)*, response to stress (*Carhsp1*, *Sod2*, *Ahr*, *Hmox1*), among others (*Srpk1*). Complementary, Ingenuity-based analysis showed that the top-scoring gene networks differentially modified by HP1γ knockdown were related to cellular development, gene expression, and cell cycle. For instance, a representative example of this type of gene networks, shown in Fig. 4d, pertains to regulation of the Wnt signaling pathway, which has widely been implicated in the promotion of proliferation and unipotent properties of spermatogonial stem cells [20]. Therefore, we conclude that our genome-wide data obtained though the knockdown of HP1γ in male germ cell lines is congruent with a role for this protein in male germ cell division, as supported by both our immunochemical analyses and mechanistic cell biological experiments.

### Phosphorylation at Ser^83^ plays a role in HP1γ-mediated regulation of spermatogenesis-associated gene expression networks

To characterize the relationship of HP1γ phosphorylation on the regulation of genes identified by HP1γ knockdown, we performed a rescue experiment by expressing wild type HP1γ or phosphorylation mutants. Toward this end, we utilized our GC1-HP1γ knockdown cells and transduced them with adenoviral vectors expressing empty vector (EV), wild type HP1γ (WT-HP1γ), the non-phosphorylatable mutant (HP1γ-S83A), or a phosphomimetic form of Ser^83^-HP1γ (HP1γ-S83D) for Affymetrix whole genome gene expression analysis (Additional file 3: Table S6). Genes that were not significantly regulated by WT-HP1γ, HP1γ-S83A, or HP1γ-S83D (p > 0.95, fold change ±2, adjusted to EV expression) were compared to genes significantly altered in the presence of *HP1*γ knockdown (p < 0.05, fold change ±1.25). Rescue was *a priori* defined as a significant reversal in expression of the gene loci identified by HP1γ knockdown in the presence of either wild type or phospho-mutant HP1γ (S83A or S83D). Of the 273 genes affected by HP1γ knockdown identified in the previous experiment (Fig. 4a), 79 genes were not rescued by WT-HP1γ or either mutant (Additional file 4: Table S2), which suggests that their expression is not directly modulated by HP1γ or is an artifact of the gene knockdown. Expression of the phosphomimetic (S83D) and the non-phosphorylatable (S83A) forms rescued 77 genes (39.69 %; Additional file 4: Table S2), indicating that a significant portion of HP1γ function in these cells is dependent on phosphorylation. Notably, both mutants altered expression of a large subset of genes not identified in the knockdown rescue unique from wild type HP1γ overexpression, suggesting that mutation of the serine 83 site and altered phosphorylation status may possess profound pathway disruption effects. Additionally, 117 genes were rescued by WT-HP1γ (43 %; Additional file 4: Table S2). As the serine 83 site on the wild type HP1γ molecule is intact, the dependency of phosphorylation on the rescue of these genes is possible but indeterminate. From these data, we conclude that the expression of a subset of spermatogenesis-associated genes identified by HP1γ knockdown requires not only the expression but also the phosphorylation of this protein for their transcriptional control.

**Fig. 5:**
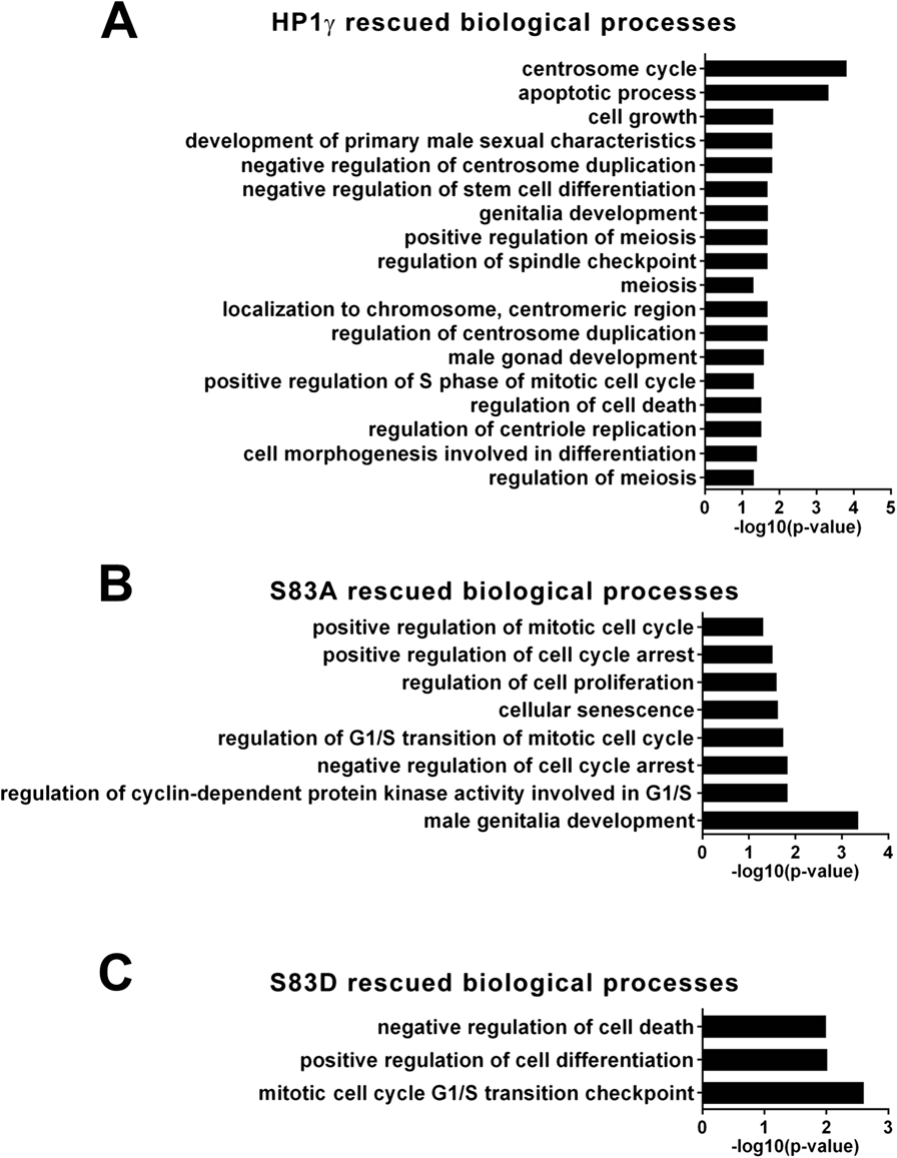
Rescue of mitosis and meiosis processes mediated by HP1γ is dependent on its phosphorylation status. **a-c**. Gene Ontology (GO) ANOVA analysis of rescued targets was performed and revealed significant (p < 0.05) enrichment of mitosis and meiosis associated processes, as well as processes involved in cellular expansion and differentiation for **(a)** HP1γ, **(b)** HP1γ and HP1γ-S83A but not HP1γ-S83D or **(c)** HP1γ and HP1γ-S83D but not HP1γ-S83A. Targets, rescued by the wild type protein but not one of the mutants, indicate targets explicitly dependent on HP1γ phosphorylation or dephosphorylation

To gain better insight into how HP1γ phosphorylation status affects spermatogenesis-associated gene networks, we performed gene enrichment ontological analysis of gene targets rescued by WT and the phosphorylation mutants (Fig. 5a-c). Accordingly, we found that WT-HP1γ rescued genes involved in various aspects of mitosis, including spindle checkpoint, protein localization to the centrosome, centriole replication, and centrosome duplication (Fig. 5a). Various processes related to morphogenesis were significantly enriched, such as meiosis, apoptosis, and cellular differentiation. Processes rescued by the S83A mutant, but not the S83D mutant, included G1/S regulation, as well as processes involved in delays or arrest of mitosis, indicating a requirement for HP1γ dephosphorylation during these events (Fig. 5b). Targets rescued by the S83D mutant, which were surrogates for genes which their expression requires HP1γ phosphorylation, participate in mitotic G1/S checkpoint as well as cellular differentiation (Fig. 5c). A number of signaling cascades displayed enrichment with both mutants (Additional file 5: Table S3, Additional file 6: Table S4, Additional file 7: Table S5), including Wnt, RAS, ERK, MAPK, and TNF, signifying a requirement for HP1γ phosphorylation in the regulation of gene networks that support differentiation, growth, and survival processes during spermatogenesis [20–24]. Taken together, these results support a role for HP1γ in cell cycle processes intrinsic to the expansion and differentiation of germ progenitor cells in a manner that is highly dependent on the Ser^83^ phosphorylation status of this protein.

## Discussion

This study demonstrates the importance of HP1γ and its phosphorylated Ser^83^ form in gametes and early embryonic development. Here, we describe for the first time the temporal and spatial relationship of HP1γ with gametes and the preimplantation embryo. P- Ser^83^-HP1γ is present at the centriole and mid-piece of the mature spermatozoa and lacking at the spindle pole of the M2 oocyte. After fertilization, this protein resumes its mitotic role [10], as well as its euchromatic interphase localization upon mouse early embryonic genome activation at the 1 to 2-cell transition. The concerted regulation of relevant gene networks further confirms the importance of HP1γ in fertility.

HP1γ and its P-Ser^83^ form are localized to highly proliferating cells of the testis. HP1γ is present in both mitotic and meiotic cell populations with high levels localizing to spermatogonia and spermatocytes, whereas the P-Ser^83^ form is preferentially located in mitotically active spermatogonia, indicating the importance for the modification of this protein specifically during mitotic divisions. The regulation of relevant gene networks further confirms the importance of this protein and its Ser^83^ phosphorylation during spermatogenesis, as mitotic, meiotic, cellular proliferation, and cellular differentiation processes are dependent on HP1γ phosphorylation status. Thus, our data provide evidence that the appropriate transmission of HP1γ-mediated epigenetic signals is necessary for normal spermatogenesis and suggest that its disruption may contribute to problems with male fertility.

Using immunofluorescence and electron microscopy, we also localized P- Ser^83^-HP1γ to the midpiece of the spermatozoa, where it is highly concentrated in the centriole. Although murine sperm do not contribute their centrosome during fertilization [25], interestingly, studies in human Intra Cytoplasmic Sperm Injection (ICSI) have demonstrated that the intact axoneme along with the sperm head carrying the genetic material are required for successful fertilization [26]. Furthermore, embryo development with this procedure and embryos resulting from injection of dissected sperm revealed chromosomal mosaicism with lack of bipolar spindle [26]. Therefore, it would be interesting to see what role, if any, this modified form of HP1γ and its association with the centriole plays in this process.

The current study focused primarily on the pre-meiotic spermatogenic cell population, for which we observed the highest P-Ser83-HP1γ levels, by utilizing GC1, a SV40-immortalized mouse Type B spermatogonia cell line with characteristics of a stage between type B spermatogonia and primary spermatocytes. Our previous work has shown mitotic aberrations, cells with multiple centrosomes, and irregular spindle poles in somatic cells with HP1γ knockdown [10]. Here, we confirm these findings in the GC1 cell line by demonstrating a significantly elevated number of abnormal mitoses in the knockdown group when compared with control. This protein was not present at the spindle poles of M2 oocytes that were awaiting fertilization, indicating that this may be an important spermatogenic contribution to the developing embryo, in particular, during early genome activation.

This is the first study to demonstrate the relationship of HP1γ and its P-Ser83 form in mouse oocytes and early embryonic development. We demonstrate that P-Ser83-HP1γ is not present in the euchromatin of pronuclear embryos and does not take on a mitotic localization at the spindle poles until the first or second cell division. After this transition, P-Ser83-HP1γ is found in the euchromatin of interphase nuclei. PCR of mouse oocytes and early embryos supported these findings demonstrating low transcription of HP1γ in mouse oocytes and pronuclear embryos with increased transcript levels at the 1–2 cell (Day 0.5) transition. Interestingly, the 1–2 cell mouse embryonic transition has been shown to coincide with early embryonic genome activation [19], suggesting that this epigenetic regulator is immediately engaged in its role in chromatin organization, as well as subsequent cell divisions.

## Conclusions

In summary, the results of this study provide evidence that HP1γ in sperm may contribute to early embryonic mitotic divisions that occur at the time of embryonic genome activation. Furthermore, disruption of this HP1γ-Aurora Kinase pathway may contribute to spermatogenic as well as early embryonic cleavage abnormalities leading to infertility or early pregnancy loss.

## Additional files

Additional file 1: Fig. S1. qPCR validation of Affymetrix data. qPCR analysis was performed on GC-1 *HP1*γ knockdown cells with adenoviral transduction of empty vector (EV), wild type HP1γ, HP1γ-S83A, or HP1γ-S83D. For the purposes of validation, genes were considered significantly regulated if p < 0.05 for Affymetrix. Fold change for each condition (WT, S83A, S83D) adjusted to EV expression is represented on a scale of ±2 and shown next to the corresponding Affymetrix data.

Additional file 2: Table S1. GO ANOVA of *HP1*γ knockdown in male germ cells.

Additional file 3: Table S6. Master processed data file from Affymetrix gene expression arrays with all of the genes and fold changes.

Additional file 4: Table S2. Rescue of the 273 genes affected by HP1γ knockdown. Table indicates Gene Symbol, Reference Sequence (RefSeq) number, as well as whether the gene was rescued by wild type (WT), S83A, and/or S83D HP1γ , or not rescued at all.

Additional file 5: Table S3. Gene ontology enrichment identification of biological processes associated with HP1γ rescue.

Additional file 6: Table S4. Gene ontology enrichment identification of biological processes associated with HP1γ and HP1γ-S83A rescue.

Additional file 7: Table S5. Gene ontology enrichment identification of biological processes associated with HP1γ and HP1γ-S83D rescue.
