# Supplementary figures and images for: The Aurora A-HP1γ pathway regulates gene expression and mitosis in cells from the sperm lineage
Source: BMC Dev Biol. 2015 May 29;15:23. doi: 10.1186/s12861-015-0073-x (PMC4448908; doi:10.1186/s12861-015-0073-x)

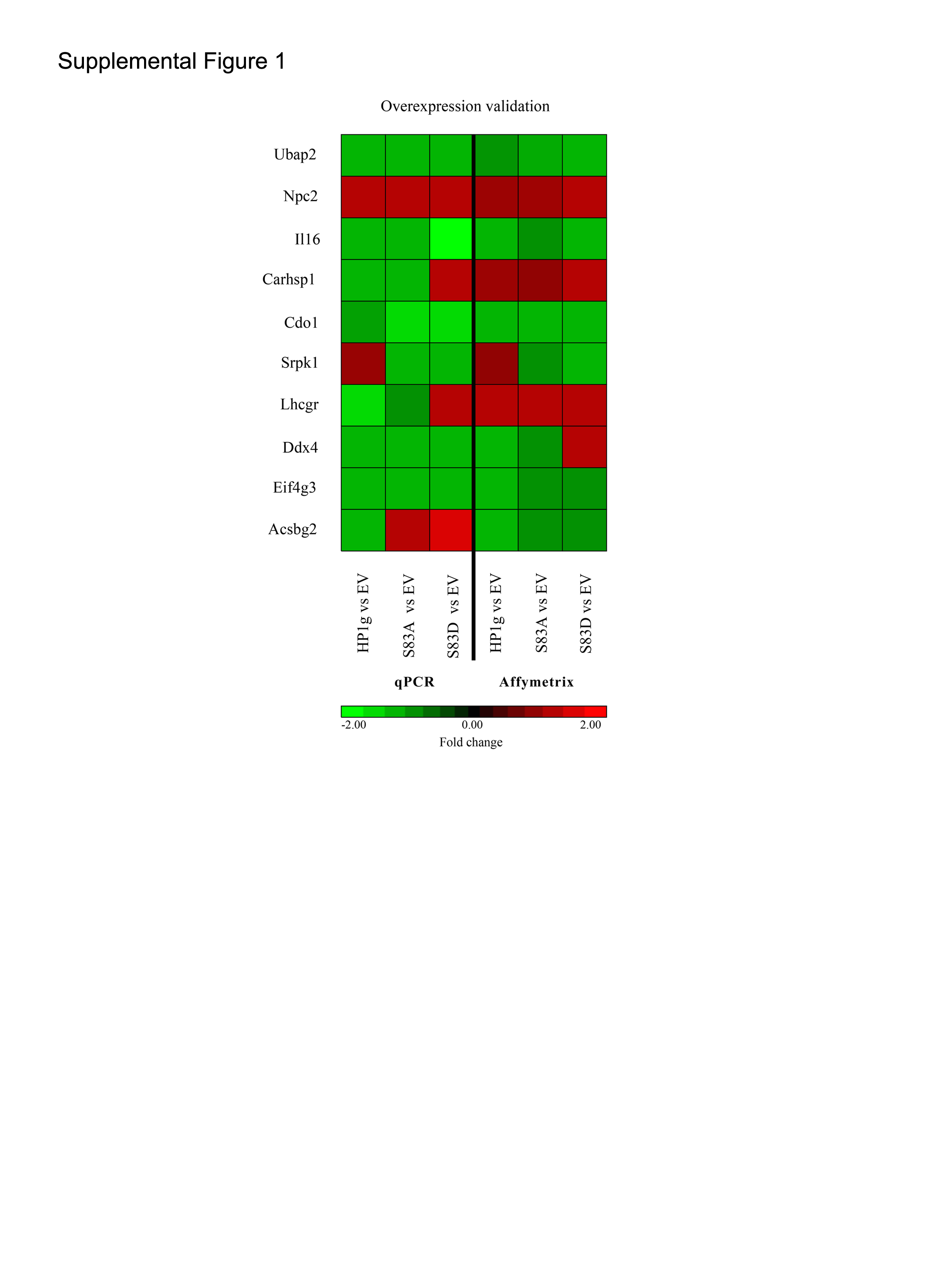

Supplement: Supplementary file 1 — qPCR validation of Affymetrix data. qPCR analysis was performed on GC-1 HP1γ knockdown cells with adenoviral transduction of empty vector (EV), wild type HP1γ, HP1γ-S83A, or HP1γ-S83D. For the purposes of validation, genes were considered significantly regulated if p < 0.05 for Affymetrix. Fold change for each condition (WT, S83A, S83D) adjusted to EV expression is represented on a scale of ±2 and shown next to the corresponding Affymetrix data. [file 12861_2015_73_MOESM1_ESM.png]
